# The Energy Metabolism Dysfunction in Psychiatric Disorders Postmortem Brains: Focus on Proteomic Evidence

**DOI:** 10.3389/fnins.2017.00493

**Published:** 2017-09-07

**Authors:** Giuliana S. Zuccoli, Verônica M. Saia-Cereda, Juliana M. Nascimento, Daniel Martins-de-Souza

**Affiliations:** ^1^Laboratory of Neuroproteomics, Department of Biochemistry and Tissue Biology, Institute of Biology, University of Campinas Campinas, Brazil; ^2^Instituto Nacional de Biomarcadores em Neuropsiquiatria (INBION), Conselho Nacional de Desenvolvimento Cientifico e Tecnologico São Paulo, Brazil

**Keywords:** proteome, schizophrenia, depression, bipolar disorder, mass spectrometry

## Abstract

Psychiatric disorders represent a great medical and social challenge and people suffering from these conditions face many impairments regarding personal and professional life. In addition, a mental disorder will manifest itself in approximately one quarter of the world's population at some period of their life. Dysfunction in energy metabolism is one of the most consistent scientific findings associated with these disorders. With this is mind, this review compiled data on disturbances in energy metabolism found by proteomic analyses of postmortem brains collected from patients affected by the most prevalent psychiatric disorders: schizophrenia (SCZ), bipolar disorder (BPD), and major depressive disorder (MDD). We searched in the PubMed database to gather the studies and compiled all the differentially expressed proteins reported in each work. SCZ studies revealed 92 differentially expressed proteins related to energy metabolism, while 95 proteins were discovered in BPD, and 41 proteins in MDD. With the compiled data, it was possible to determine which proteins related to energy metabolism were found to be altered in all the disorders as well as which ones were altered exclusively in one of them. In conclusion, the information gathered in this work could contribute to a better understanding of the impaired metabolic mechanisms and hopefully bring insights into the underlying neuropathology of psychiatric disorders.

## Introduction

Psychiatric disorders represent a great medical and social challenge and people suffering from those conditions face many impairments regarding personal and professional life. Many of those disorders have an onset on early adult life and can severely affect a person's well-being and ability to be functional (Hyman, 2008). Approximately one quarter of the world's population will manifest a mental disorder at some period of their life (World Health Organization, 2008b), and people affected by psychiatric disorders have a mortality rate 2.22 times higher than the general population, with a 10-year shorter lifespan (Walker et al., 2015). Worldwide, 4 out of 10 estimated causes of disability are neuropsychiatric disorders, and among the most prevalent are schizophrenia (SCZ), bipolar disorder (BPD), and major depressive disorder (MDD) (World Health Organization, 2008b). Despite the great burden related to psychiatric disorders and the extensive research made on the topic, the mechanisms and risk-factors associated with them have not yet been elucidated.

Over the years, the brain has been extensively studied in attempts to understand these disorders, searching for possible causes and treatments. SCZ, BPD, and MDD are characterized as disorders brought on by small flaws in several brain areas rather than a greater damage in an individual brain region. This implies that those disorders could derive from flawed connections between components of the neural system (Fornito and Harrison, 2012). The recurring presence of metabolic alterations related to energy pathways has been suggested as one of the most important physiological features of SCZ, BPD, and MDD (Shao et al., 2008).

The human brain constitutes 2% of the whole body weight and, paradoxically, is responsible for ~25% of total body glucose utilization (Bélanger et al., 2011). Glucose is the obligatory energy substrate of the brain and it goes through many reactions in order to produce adenosine triphosphate (ATP) through sequential processing by glycolysis, the tricarboxylic acid (TCA) cycle, and oxidative phosphorylation (OXPHOS) (Magistretti, 2004). Oxidative metabolism is an crucial process in maintaining cell viability as it generates great amounts of ATP, however, this accelerated rate of oxidation within the cell culminates in the production of potentially detrimental by-products, called reactive oxygen species (ROS) (Magistretti, 2008). These highly ROS, if not neutralized by the action of antioxidant enzymes, can cause damage to carbohydrates, lipids, proteins, and DNA, potentially resulting in functional deficits and even cell death (Manji et al., 2012).

Glucose also plays an important role in entering the metabolic pathways that lead to the synthesis of glutamate, acetylcholine, gamma-aminobutyric acid (GABA), all three being key neurotransmitters (Deutch and Roth, 2004; Magistretti, 2004). Mitochondria, which are responsible for playing an important role in cellular energy generation, also promotes calcium buffering, ROS neutralization (Clay et al., 2011) and are intimately involved with amino-acid metabolism. The brain's high energy demand is mainly due to the myriad of energy-intensive processes, including axonal action potentials, cell signaling, presynaptic Ca^2+^ entry, uptake and recycling of neurotransmitters and synaptic vesicle releasing (Attwell and Laughlin, 2001; Alle et al., 2009). In regions of gray matter, there is a majority of excitatory synapses compared to inhibitory synapses, suggesting that excitatory neurotransmission accounts for most of the energy demands at the cortical level (Bélanger et al., 2011). Depending on the activity performed at the time, energy consumption in the related brain region is stimulated, and for that reason there is an increase on blood flow to that particular area, since energy substrates reach their targets by the circulatory system (Magistretti and Allaman, 2013).

The possibility to study postmortem brain tissue derived from patients with psychiatric disorders have provided valuable insights into the physiopathology of those disorders, since the brain is rarely if ever accessible for biopsies from living patients. Many studies have been conducted on postmortem brain tissue from patients suffering from neuropsychiatric disorders. Proteomic studies have the advantage to provide valuable information on which proteins were present at the time of the course of illness and their expression level (Bayés and Grant, 2009; Gottschalk et al., 2015). Given the importance of energy metabolism in the brain and its important role in the pathophysiology of neuropsychiatric disorders, this review aims to compile data on energy metabolism dysfunction found on postmortem brain tissue revealed by proteomics in SCZ, BPD, and MDD.

## Psychiatric disorders and evidences of overall metabolic dysfunction

### Schizophrenia

SCZ is known to affect ~1% of the world population. As a complex syndrome thought to be of neurodevelopmental origin (Rapoport et al., 2005), is manifested through a wide range of severe symptoms, and patients experience a combination of what are classified as positive, negative, and cognitive symptoms. Positive symptoms are related to the loss of touch with reality, represented by auditory hallucinations and delusions, negative symptoms include the inability to feel pleasure, flattening of affect, and social withdrawal. Cognitive dysfunction is also an important characteristic of schizophrenia and includes a decreased ability to focus as well as attention and memory deficiencies (Wood and Freedman, 2003). The onset of the illness, often not recognized as such, begins with decline in cognitive and social functioning in early adolescence and precedes the onset of psychosis by ~10 years (Kahn et al., 2015). SCZ is thought to be one of the most severe mental disorders and the average life expectancy is ~20 years below that of the general population (Laursen et al., 2014).

Blood sample analyses of antipsychotic SCZ patients have detected elevated levels of insulin (Guest et al., 2010) and increased insulin resistance (Venkatasubramanian et al., 2007; van Nimwegen et al., 2008). In addition, there was a higher prevalence of hyperglycemia and impaired glucose tolerance in schizophrenia patients when compared to healthy controls (Ryan et al., 2003; Spelman et al., 2007; Fernandez-Egea et al., 2008, 2009).

Many investigators have found correlations between the occurrence of psychosis and altered blood flow and metabolism in different brain regions (Cleghorn et al., 1989; Gur et al., 1989; Andreasen et al., 1992; Siegel et al., 1993). Unmedicated patients with schizophrenia were studied with ^18^F-fluorodeoxyglucose positron emission tomography (PET) and magnetic resonance imaging (MRI) to evaluate glucose metabolism and to obtain volumetric measurements, respectively. This study revealed that, when compared to controls, SCZ patients displayed lower relative glucose metabolic rates and volumetric reductions in an area of the cingulate gyrus related to higher executive functions (Haznedar et al., 2004). In a similar study that focused on three thalamic nuclei, it was observed that reduced relative glucose metabolism in the pulvinar nucleus was associated with more hallucinations and positive symptoms, while metabolic reductions in the mediodorsal nucleus were associated predominantly with negative symptoms (Hazlett et al., 2004). There have also been reports of significantly lower levels of pyruvate in the mediodorsal thalamus of patients with SCZ (Martins-De-Souza et al., 2010a).

Schizophrenia has also been associated with mitochondrial dysfunction and the presence of mutations and polymorphisms in mitochondrial (Rollins et al., 2009; Clay et al., 2011). Mitochondrial hypoplasia has also been observed (Uranova et al., 2001) in addition to significant alterations in the enzymatic activity of Complex I located in the mitochondrial inner membrane, which together point to a dysfunction of the oxidative phosphorylation system (Dror et al., 2002) and decreased ATP production (Volz et al., 2000) in schizophrenia patients. Disturbances due to oxidative stress disturbance are evident in schizophrenia, such as the higher activity levels of superoxide dismutase (SOD) and glutathione peroxidase (GSH-Px) show higher activity levels when compared to healthy controls (Mahadik et al., 2001; Kuloglu et al., 2002). Another important cellular process related to mitochondria is the maintenance of calcium homeostasis, and studies have shown impairment of calcium homeostasis and signaling in schizophrenia (Bojarski et al., 2010). All the aforementioned processes altered in schizophrenia are implicated in synaptic remodeling, and their dysfunction may induce a wide range of harmful effects, and consequently affect brain plasticity (Martins-de-Souza et al., 2011).

### Major depressive disorder

MDD is a heterogeneous, debilitating, and at times, life-threatening psychiatric disorder that affects ~350 million people worldwide (World Health Organization, 2008a) and the lifetime incidence of depression is more than 12% in men and 20% in women (Kessler et al., 2003). Patients with MDD present a lasting feeling of sadness or irritability and, in addition, can present several psychological and physiological disturbances, such as flattening of affect, reductions in appetite and libido, suicidal thoughts and slowing of speech and action (Belmaker and Agam, 2008).

Some factors related to metabolic syndrome including obesity, diabetes, and hyperglycemia have been associated with the presence of depression; and there are also reports of insulin resistance in MDD patients (Everson-Rose et al., 2004; Skilton et al., 2007).

PET measurements from MDD patients revealed a reduction in both blood flow and glucose metabolism in the caudate nucleus, anterior cingulate cortex and prefrontal cortex during tests that were conducted both in a resting state and under stressful situations (Videbech, 2000). However, analysis of the orbital cortex, medial thalamus, and amygdala displayed increased blood flow and glucose metabolism (Drevets, 2001). Another study made use of the administration of a stable isotope (^13^C) that is detected by magnetic resonance spectroscopy (MRS) to evaluate the processes associated with neurotransmission and metabolism in MDD patients. It was possible to observe that glutamatergic neurons displayed hampered TCA cycle rates when compared to controls, implicating that the glutamatergic system and mitochondrial energy metabolism may have an important role in the pathology of this disorder (Abdallah et al., 2014).

In concordance with these data, significant impairment of mitochondrial ATP production and lower activity levels of mitochondrial enzymes have been reported in MDD patients compared to controls. Additionally, an increased proportion of patients displayed deletions in mitochondrial DNA (mtDNA) indicating the presence of mitochondrial dysfunction (Gardner et al., 2003).

### Bipolar disorder

BPD is a chronic mood disorder characterized by transitions between manic and depressive episodes, which is estimated to affect up to 4% of the population (Merikangas et al., 2011). Manic episodes can be described as an overall exacerbation of emotions, such as euphoria and elevated optimism. Those characteristics, together with sleep deprivation caused by overactivity may become extreme and hamper a patient's well-being and decision-making ability (Belmaker, 2004). Individuals with BPD have high rates of disability and often experience persistent neurocognitive deficits and poor psychosocial functioning (Kapczinski et al., 2014).

Individuals with BPD present a higher incidence of metabolic syndrome in comparison to the general population (Fagiolini et al., 2005; Taylor and MacQueen, 2006; Garcia-Portilla et al., 2008). By compiling data from the prevalence of metabolic syndrome, it was observed that the rate of metabolic syndrome varied from 17 to 67% in BPD patients (Grover et al., 2012). This syndrome is a high-risk factor for cardiovascular disease and type-2 diabetes mellitus. Studies reported that individuals with BPD are more affected by hyperglycemia, type-2 diabetes mellitus and insulin resistance than the general population (Grover et al., 2012). Medical conditions that are chronic and stress-sensitive, such as cardiovascular disease, obesity, and type-2 diabetes mellitus are the most prominent causes of mortality among individuals with BPD (Brietzke et al., 2011; Vancampfort et al., 2013).

The observation of cerebral blood flow in individuals experiencing mania symptoms revealed that there was a flow decrease in different brain regions, such as the right ventral lobe and frontal regions, when compared to healthy controls (Migliorelli et al., 1993; Blumberg et al., 1999). Interestingly, in a different study it was verified that manic patients presented higher cerebral blood flow in the left dorsal anterior cingulate cortex when compared to not-manic BPD patients (Blumberg et al., 2000).

The evaluation of markers normally linked to metabolic dysfunctions revealed lower serum levels of glucagon, glucagon-like peptide-1 (GLP-1), ghrelin, and higher levels of glucose-dependent insulinotropic polypeptide (GIP) in BPD patients (Rosso et al., 2015). Glucagon is known to act on the system for psychological stress response (Perry et al., 2014). GLP-1 and GIP receptors are expressed in brain areas predominantly involved in mood and cognitive function (Alvarez et al., 2005). Therefore, these markers could be pivotal to the association between bipolar and metabolic disorders (Czepielewski et al., 2013) as they perform an important role in mechanisms of brain synaptic plasticity and neuroprotection, which were found to be altered in neuroimaging studies of BPD patients (Canales-Rodríguez et al., 2014).

Gray matter analysis of medication-free BPD patients revealed elevated levels of lactate and decreased intracellular pH in the prefrontal cortex. These characteristics suggest that cells are relying mainly on glycolysis rather than OXPHOS to acquire energy, which in turn may indicate that mitochondrial functionality is hampered in BPD (Dager et al., 2004; Weber et al., 2013). Abnormalaties in mitochondrion structure and mutations and polymorphisms in mitochondrial DNA (mtDNA) have been reported in patients with BPD (Shao et al., 2008; Cataldo et al., 2010), which could compromise the integrity and functionality of mitochondria, the efficiency of OXPHOS, the Ca^2+^ buffering, and neutralization of ROS in BPD (Clay et al., 2011).

## Evidence of compromised energy metabolism in neuropsychiatric disorders revealed by proteomics

Proteomics has the goal to obtain a global view of the proteins present in a given cell or tissue at a determined moment and state; only this snapshot is possible because the proteome is dynamic, with different proteins being constantly degraded and produced in response to various internal and external stimuli (Graves and Haystead, 2002). The proteome represents the genetic information that has been transcribed and translated, after any modifications at the epigenetic, mRNA, and post-translational levels (Nascimento and Martins-de-Souza, 2015). By understanding the information obtained from these studies, it has been proposed that proteomics may provide more accurate information about the pathophysiology of a disease than other approaches such as genomics and transcriptomics, as it represents what proteins are present at any important moment during the course of the illness (Bayés and Grant, 2009; Gottschalk et al., 2015). Therefore, mass spectrometry (MS)-based proteomics methods have been widely used in several studies, as they have the ability to identify, as well as quantify innumerous disease-associated protein changes in a given sample (Föcking et al., 2014).

### Proteomic techniques

Proteomic methods employed in the study of neuropsychiatric disorders began with the development of two-dimensional gel electrophoresis (2DE) (O'Farrell, 1975). By late 1990s it was developed the differential two-dimensional electrophoresis (2D-DIGE) (Unlu et al., 1997). The major limitation of the 2DE and 2D-DIGE techniques is the separation of proteins with more extreme characteristics, including those that are hydrophobic, too large or too small, or extremely basic or acidic. Despite the limitations, these techniques represent a very high-quality top-down method of total proteome resolution, resolving protein isoforms and post-translational modifications (O'Farrell, 2008; Oliveira et al., 2014).

In 1999, a technique was described to perform protein identification by first using liquid chromatography (LC) and then tandem mass spectrometry (MS/MS) to separate and fragment peptides. From this, the term “shotgun proteomics” was coined (Link et al., 1999). This approach is under continuous development to achieve a better coverage of a sample's whole proteome. Considering recent developments, proteomic studies consist of the analysis of a digested proteome, which goes through chromatographic separation, of one or more dimensions, followed by MS/MS analysis (Aebersold and Mann, 2003; Taylor et al., 2009).

Due to methods developed using mass-spectrometry based approaches for quantitative proteomics, currently is possible to monitor global protein expression and to obtain important quantitative data (Ong et al., 2003).

## What can proteomics tell us about energy metabolism dysfunction?

This review searched and analyzed every postmortem brain tissue-based proteomic work published so far regarding patients with schizophrenia (SCZ), bipolar disorder (BPD), and MDD. We searched in the PubMed database (www.ncbi.nlm.nih.gov/pubmed) to gather the studies and compiled all the differentially expressed proteins reported in each work. The proteins were searched for individually in the Human Protein Reference Database (http://hprd.org) to determine their biological process and in which cellular component(s) they are normally present. Twenty-two articles on SCZ were found (Johnston-Wilson et al., 2000; Prabakaran et al., 2004; Beasley et al., 2006; Clark et al., 2006; Sivagnanasundaram et al., 2007; Pennington et al., 2008a,b; Behan et al., 2009; English et al., 2009; Martins-de-Souza et al., 2009a,b,c,d; Martins-De-Souza et al., 2010a,b; Föcking et al., 2011, 2014; Wesseling et al., 2014; Gottschalk et al., 2015; Saia-Cereda et al., 2015, 2016; Schubert et al., 2015), 10 on BPD (Johnston-Wilson et al., 2000; Beasley et al., 2006; Pennington et al., 2008a; Behan et al., 2009; Föcking et al., 2011, 2016; Wesseling et al., 2014; Gottschalk et al., 2015; Schubert et al., 2015; Stelzhammer et al., 2015), and seven on MDD (Johnston-Wilson et al., 2000; Beasley et al., 2006; Martins-de-Souza et al., 2012a,b; Wesseling et al., 2014; Gottschalk et al., 2015; Stelzhammer et al., 2015). After annotating biological processes and cellular components, we selected those that were related to metabolism and energy pathways and compiled this data in with information on up- or down-regulation, when available, from which specific brain region was the postmortem tissue and what was the proteomic technique used in the study (Supplementary Table 1). SCZ studies revealed 92 differentially expressed proteins related to energy metabolism, while 95 proteins were discovered in BPD and 41 proteins in MDD (Supplementary Table 1). Information regarding sample size, gender, age, drug treatment, and brain pH from each study compiled on SCZ, BPD, and MDD can be found in the Supplementary Table 2.

It is important to highlight that all the studies mentioned above have been performed using brain tissue collected from patients treated with a wide range of drugs and for that reason it cannot be ruled out that at least some findings could be attributed to a drug-derived artifact rather than the disorder itself. However, as the results will be discussed in the upcoming section, this potential bias could be elucidated as there are evidences suggesting that alterations of energy metabolism described in SCZ, BPD, and MDD are a component of the diseases themselves and not an effect of the treatments used.

### Similarities among SCZ, BPD, and MDD

**Figure 1:**
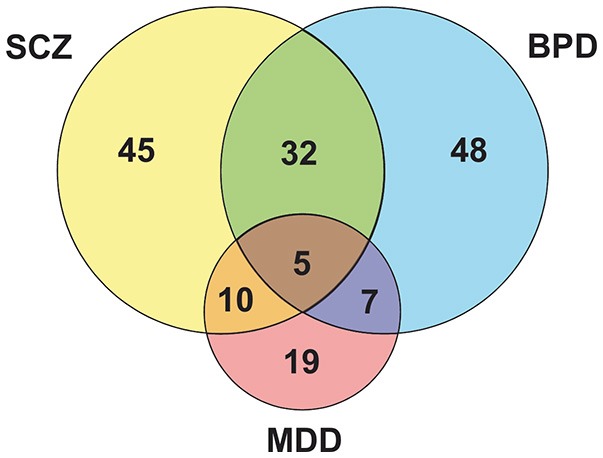
Venn diagram evidencing peculiarities and similarities among the major psychiatric disorders.

**Figure 2:**
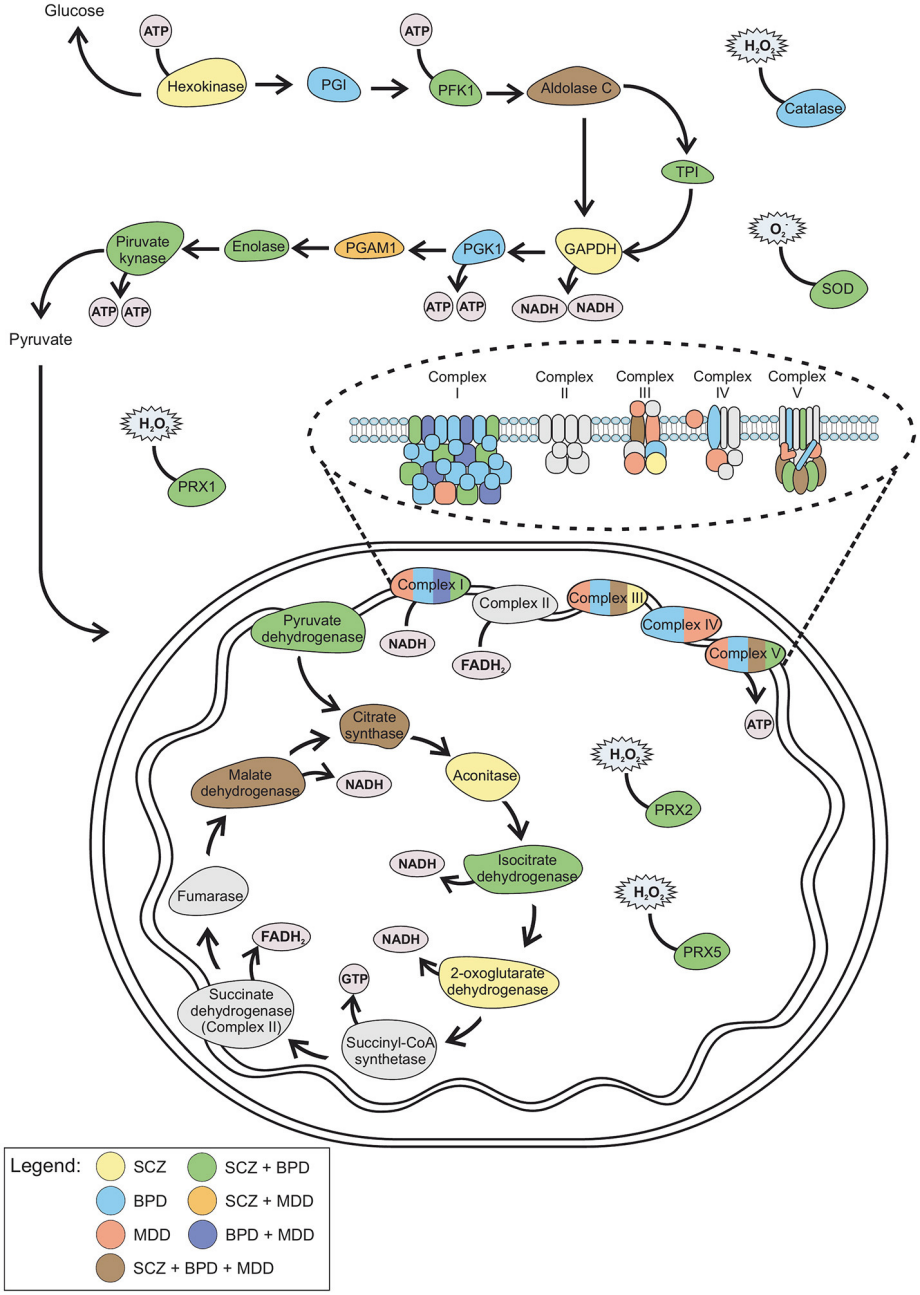
Schematic representation of the altered proteins in SCZ, BPD, and MDD. Color from each disorder and combination of disorders correlates with the Venn diagram (Figure 1). GAPDH, Glyceraldehyde 3-phosphate dehydrogenase; PFK1, Phosphofructokinase 1; PGAM, Phosphoglycerate mutase; PGI, Phosphoglucose isomerase; PGK1, Phosphoglycerate kinase 1; PRX, Peroxiredoxin; SOD, Superoxide dismutase; TPI, Triosephosphate isomerase.

Five proteins overlapped as differently expressed in all three disorders (Figure 1 and Supplementary Table 1): aldolase C, citrate synthase, malate dehydrogenase, cytochrome bc1 core protein 1, and ATP synthase subunit beta (Figure 2). Aldolase C is a crucial enzyme in glycolysis responsible for the conversion of fructose-1,6-bisphosphate to glyceraldehyde-3-phosphate and dihydroxyacetone phosphate. Citrate synthase is a key enzyme of the TCA cycle and catalyzes the reaction in which citrate is formed by the condensation of the acetate residue from acetyl-CoA with oxaloacetate. Malate dehydrogenase is another enzyme of the TCA cycle and catalyzes the NAD^+^/NADH-dependent interconversion of the substrates malate and oxaloacetate. Cytochrome bc1 core protein 1 is located within the mitochondrial matrix and the full cytochrome bc1 complex is a key component of the respiratory electron transport chain embedded in the inner membrane of mitochondria. The beta subunit of ATP synthase is the portion that is responsible for the conversion of ADP to ATP, which occurs due to the proton gradient across the membrane formed by OXPHOS reactions.

These differences involve the main axis of metabolic pathways for ATP production, with a slight focus on oxidative phosphorylation. The common differential expression of citrate synthase and malate dehydrogenase may hypothetically connect these disorders to lipid production impairment. Citrate synthase is downregulated in SCZ and upregulated in BPD and MDD whereas malate dehydrogenase in downregulated in BPD and upregulated in SCZ and MDD. This disruption in ATP production may switch the metabolic demand of the cell to obtain energy by lipid breakdown in the brain.

Phospholipase A2 (PLA2), which catalyzes the cleavage of membrane phospholipids, was found to have increased activity levels in the blood of SCZ patients (Gattaz et al., 1987; Gattaz and Brunner, 1996; Ross, 1997). There have been reports of increased phospholipid turnover rates in the thalamus and frontal lobe (Gattaz and Brunner, 1996) and lower levels of docosapentaenoic acid (EPA) and phosphatidylcholine in the hippocampus of SCZ patients (Hamazaki et al., 2010). In addition, there is evidence of lower levels of arachidonic acid (AA) in erythrocytes and brain tissue of SCZ patients (Horrobin, 1996; Laugharne et al., 1996).

There have been reports of elevated rates of hydrolysis for serum phospholipids in BPD (Lieb et al., 1983; Hibbeln et al., 1989) and increased levels of prostaglandins -compounds derived from AA metabolism- in serum, saliva and cerebrospinal fluid from BPD patients, suggesting dysregulated AA metabolism (Lieb et al., 1983; Linnoila, 1983; Nishino et al., 1989). Also, upregulation of calcium-dependent cytosolic phospholipase A2 (cPLA2) was reported, an enzyme involved in AA metabolism, as well as lower concentrations of AA in the frontal cortex of BPD patients (McNamara et al., 2008; Rapoport, 2008). Further studies should be conducted in order to evaluate larger sample sizes, taking into consideration any potentially confounding factors like the dietary profile of patients and their general health status (Igarashi et al., 2010).

Cholesterol located in the myelin sheath that surrounds axons is effectively immobilized because of the slow turnover of myelin. Studies conducted on mood disorder patients revealed lower cholesterol levels when compared to controls (Beasley et al., 2005). A significant association between AA to EPA ratios present in erythrocytes and severity of depression has been stablished (Adams et al., 1996). Compared with healthy controls, patients suffering from MDD displayed significantly higher serum levels of PLA2 activity (Noponen et al., 1993) and the mRNA expression of PLA2 was significantly increased when compared with healthy controls (Mueller et al., 2015).

Several studies have shown the effects of antidepressants, antipsychotics, and mood-stabilizers on PLA2 activity (Gattaz et al., 1987; Bosetti et al., 2003; Tavares et al., 2003). The antipsychotic drug clozapine was reported to elevate the erythrocyte levels of AA and docosahexaenoic acid (DHA) in SCZ patients (Glen et al., 1996). This could indicate an additional mechanism that contributes to the therapeutic effects of clozapine (Horrobin, 1998). Lithium, at therapeutic concentrations, was shown to strongly inhibit PLA2 activity (Horrobin and Bennett, 1999).

This reveals a connection between serious psychiatric disorders—SCZ, MDD, and BPD—which have a different range of debilitating symptoms and prognosis, yet show similar alterations in energy metabolism processes. Several key components of the three pivotal processes in energy metabolism are altered in these disorders, which highlights the importance of proper functioning of how the brain handles energy production. Nevertheless, is important trying to assess whether brain metabolism dysfunction is a cause or consequence in the establishment of psychiatric disorders. Reports have shown that people suffering from mitochondrial diseases frequently show psychiatric symptoms such as psychosis, depression, personality change, and BPD (Manji et al., 2012). In fact, major depression has been described as being the initial symptom of mitochondrial disease in a large sample size of adult patients (Fattal et al., 2007). Mitochondrial function and energy metabolism were shown to play an important role in regulating social behaviors (Hollis et al., 2015). In addition, limited energy production may impair adaptive neuronal capacity and contribute as one of the causes to the development of psychopathologies such as SCZ, BPD, and MDD under stressful stimulus (Koene et al., 2009).

### Similarities between disorders

Data show that SCZ and BPD share 32 altered proteins (Figure 1 and Supplementary Table 1), which are mostly related to mitochondrial electron transport, response to ROS and glycolysis (Figure 2). Even though SCZ and BPD represent two distinct types of psychiatric disorders, the first being of thought or cognition and the second being of emotion, they share some pathophysiological traits such as a chronic and relapsing illness trajectories (Iwamoto et al., 2005). Alterations in components of the electron transport chain, such as subunits from NADH dehydrogenase, or glycolytic enzymes, as pyruvate kinase and phosphofructokinase, reveal an overall energy metabolism dysregulation that may relate to dysfunctions in mitochondrial processes. Oxidative damage to the brain may be partially responsible for the pathophysiological process in BPD and SCZ (Wang et al., 2009).

Oxygen and ROS metabolism pathways were found to be significantly increased, which indicates the presence of increased levels of ROS and oxidative stress generation in SCZ patients (Prabakaran et al., 2004), and the level of tyrosine nitration, which reflects the level of endogenous ROS, was significantly higher in BPD patients than in unaffected controls (Kunz et al., 2008). As was previously explored, disturbances in lipid metabolism are present in SCZ and BPD and this could, in turn, hypothetically contribute to the establishment of oxidative stress establishment since ROS are a natural by-product of lipid metabolism (Martins-de-Souza et al., 2011). With this is mind, is important to stress that further studies ought to be conducted to confirm the relation between oxidative stress and membrane phospholipid breakdown in consequence of impaired energy metabolism.

Our results showed that enzymes such as peroxiredoxins (1,2,5,6), glutathione S-transferase and superoxide dismutase are involved in protecting the cell against oxidative damage and were found to be altered in the disorders (Martins-de-Souza et al., 2009a). In situations where free radical formation surpasses the cell's antioxidant defense capacity, oxidative stress may cause direct injuries to cellular lipids, DNA and proteins, thus affecting proper cellular functioning (Cochrane, 1991). It has been proposed that oxidative damage to the brain may contribute to some extent to the development of these disorders, and associating compounds with antioxidative properties with existing treatment may be a possible approach to complement pharmacological treatment of SCZ and BPD (Wang et al., 2009).

The enzyme creatine kinase B was found to be differentially regulated in SCZ and BPD when compared to controls. This enzyme catalyzes the reversible transfer of phosphate between ATP and creatine, generating phosphocreatine (Hemmer and Wallimann, 1994). Creatine is taken up by neurons and oligodendrocytes by creatine transporters and the circuit of converting creatine to phosphocreatine by creatine kinase acts as a bioenergetic sensor that rapidly reloads ATP in the area to maintain stable levels when there are significant energy demands (Wyss and Schulze, 2002; Allen, 2012). There have been reports of decreased brain phosphocreatine levels in BPD patients in the depressed state, as compared with normal controls (Manji et al., 2012). In SCZ, levels of phosphocreatine were found to be asymmetrical in the temporal lobe of patients and lower levels of phosphocreatine were observed in the frontal brain region of patients and their first-degree relatives (Klemm et al., 2001). For that reason, decreases of phosphocreatine and ATP reported in patients with psychiatric disorders reinforces the importance of impaired energy production in those conditions (Kato, 2006).

SCZ and MDD share 10 altered proteins (Figure 1 and Supplementary Table 1), such as phosphoglucomutase 1 (Figure 2) which is an enzyme involved in glycolysis and succinyl-CoA:3-ketoacid CoA transferase which is a key enzyme in ketone body catabolism. Carbonic anhydrase I and II were also altered both in MDD and SCZ. Carbonic anhydrase I and II are expressed in erythrocytes and glial cells, respectively (Hayes, 1994). Carbonic anhydrase II is also present in myelin and the choroid plexus (Hayes, 1994) and represents one of the core determining factors of pH fluxes in neural cells (Chesler and Kaila, 1992). In schizophrenia patients, treatment with acetazolamide, which inhibits the action of carbonic anhydrase, promoted an increase in blood flow throughout the brain (Taylor et al., 1999).

Data analysis revealed seven proteins altered both in BPD and MDD (Figure 1 and Supplementary Table 1), while five of those are different subunits of the NADH dehydrogenase complex in the electron transport chain (Figure 2). This is consistent with previous reports of impaired functioning of OXPHOS complexes in MDD (Ben-Shachar, 2009; Moylan et al., 2012) and decreased nuclear expression of genes coding for mitochondrial respiratory mechanisms in BPD (Konradi et al., 2004) both of which lead to reduced mitochondrial energy production. Peroxiredoxin 5 was also altered in both disorders, which may be evidence of an increase in ROS due to poor mitochondrial functioning (Manji et al., 2012). Antidepressants used in the treatment of MDD, along with lithium which is generally used in BPD treatment, have an effect on the upregulation of mitochondrial energy generation (Scaini et al., 2011).

### SCZ exclusive

Proteins altered exclusively in SCZ (45) (Figure 1 and Supplementary Table 1) were related mostly to glycolysis and the TCA cycle (Figure 2). Hexokinase and glyceraldehyde-3-phosphate dehydrogenase are key enzymes in the handling of glucose. The alteration of these proteins along with glycolytic enzymes previously mentioned to be altered in SCZ is consistent with impaired glycolysis being an important attribute of SCZ. An approach to pharmacologically model schizophrenia in cell culture is treating cells with MK-801, which acts on the glutamatergic system. Treatment of cultured neurons, oligodendrocytes and astrocytes with MK-801 promoted a significant alteration in the level of enzymes related to glycolysis in the three cell types. Notably, of the three cell types, oligodendrocytes were the ones with more metabolic differences (Guest et al., 2015). Oligodendrocytes are glial cells responsible for neuron myelination, which is fundamental for neuronal connectivity (Davis et al., 2003) and it has been documented that oligodendrocyte dysfunction and abnormal metabolic activity are present in SCZ (Tkachev et al., 2003; Uranova et al., 2004; Bernstein et al., 2015). Most of the therapeutic targets of SCZ have been related to connectivity and synaptic transmission. Notably, clozapine is an antipsychotic drug with great clinical efficacy shown to improve glucose uptake in oligodendrocytes, indicating that in addition to rebalancing neurotransmission, this drug acts on the energy metabolism of those cells, which may in turn improve neuronal connectivity (Steiner et al., 2014; Cassoli et al., 2016). This evidence indicates SCZ may be, at least in part, a glial cell metabolic disorder, opening doors to new therapeutic targets (Bernstein et al., 2009).

Another important and informative approach in proteomics is the analysis of post-translational modifications, such as phosphorylation. While some proteins are constitutively phosphorylated, the majority present transitory phosphorylation, depending on the cellular conditions at a given time. Proteome analyses of the corpus callosum, the largest white matter structure in the human brain, rich in glial cells revealed that several proteins were differentially phosphorylated (Saia-Cereda et al., 2016). Among them was the mammalian target of rapamycin (mTOR), a kinase that is a component of the mTORC1 pathway, and it plays a role in regulating protein synthesis, mainly by direct and indirect phosphorylation (Hay and Sonenberg, 2004), as well as being an important regulator of intracellular communicatory mechanisms in glial cells (Lisi et al., 2011). The AMP-activated protein kinase (AMPK) is a cellular energy sensor and signal transducer that is regulated by a wide variety of metabolic stresses and AMPK directly phosphorylates multiple components in the mTORC1 pathway (Inoki et al., 2012). The relationship between mTOR and AMPK signaling pathways would make mTOR sensitive to even the lowest ATP depletion (Tokunaga et al., 2004). Therefore, the observation of changes in phosphorylation profile in mTOR emphasizes impaired energy production in glial cells of SCZ patients.

Transketolase and 6-phosphogluconolactonase are related to oxidation-reduction process and were altered in SCZ. They are key enzymes in the pentose phosphate pathway (PPP), which synthesizes the reduced form of nicotinamide adenine dinucleotide phosphate (NADPH) and ribose-5-phosphate (Horecker, 2002; Zhao et al., 2014). Alterations in NADPH levels and a potential imbalance in the NADP^+^/NADPH ratio have been reported in SCZ patients (Martins-De-Souza et al., 2010a). This evidence, along with the lower levels of pyruvate reported, points to glycolysis being a key pathway in the pathophysiological processes of SCZ (Martins-De-Souza et al., 2010a).

Furthermore, aconitase, isocitrate dehydrogenase, malate dehydrogenase and oxoglutarate dehydrogenase have been found to be altered in SCZ and are related to the TCA cycle. This points to alterations in mitochondrial pathways which are consistent with the concept that the broad mitochondrial processes are affected in the disorder (Ben-Shachar, 2009; English et al., 2011). Whether it is mitochondrial function or glucose metabolism that is affected first in the establishment of SCZ has yet to be elucidated (Martins-de-Souza et al., 2011).

### MDD exclusive

Proteins altered in MDD (19) (Figure 1 and Supplementary Table 1) are predominantly related to oxidative phosphorylation (Figure 2). In fact, the great majority of the proteins are subunits of cytochrome c, ATP synthase, or NADH dehydrogenase. An animal model of depression revealed that complexes from the electron transport chain were inhibited in the cerebellum and cortex when the animals were submitted to conditions of chronic mild stress (Rezin et al., 2008), while a human postmortem study of mRNA and protein levels revealed the reduced expression of three subunits of NADH dehydrogenase in the cerebellum of depressed patients (Ben-Shachar and Karry, 2008). Therefore, the poor functioning of oxidative phosphorylation due to decreased electron transport chain activity (Hroudová et al., 2013) promotes a biochemical imbalance in the processes leading to ATP production.

Mitochondrial dysfunction has been linked to depression and may be explained by deficiencies in both concentration and activity of proteins required for the proper functioning of the electron transport chain (Gardner and Boles, 2011). According to clinical studies, adults as well as children diagnosed with a primary OXPHOS disease present a higher incidence of major depression when compared to unaffected controls (Koene et al., 2009; Morava et al., 2010). Also, significant decreases of mitochondrial ATP production rates and mitochondrial enzymes ratios were observed in MDD patients (Gardner et al., 2003). Antidepressants, such as citalopram and venlafaxine promote changes in NADH dehydrogenase and cytochrome c oxidase, which indicates that those electron transport chain complexes are desirable drug targets and potential markers for MDD (Hroudova and Fisar, 2010).

### BPD exclusive

Proteins found to be altered in BPD (48) (Figure 1 and Supplementary Table 1) are mostly related to the TCA cycle and the electron transport chain (Figure 2). Microarray analyses on postmortem brain tissue revealed that several mRNAs linked to the production of mitochondrial electron transport chain complexes I–V were expressed in lower levels in BPD (Sun et al., 2006). Those findings agree with an important association between BPD and mitochondrial dysfunction (Konradi et al., 2004). As the mitochondrial electron transport chain is responsible for OXPHOS, consequently, it accounts for most of the oxygen consumption by the cell and also is responsible for substantial ROS production (Wang et al., 2009). Since polyunsaturated fatty acids, which constitute neuronal cell membranes, are very vulnerable to damage by ROS, BPD mitochondrial dysfunction may lead to overproduction of those reactive compounds, resulting in oxidative stress (Wang et al., 2009). Catalase and other previously mentioned antioxidant enzymes, such as peroxiredoxins, glutathione S-transferase and superoxide-dismutase, were found to be altered in BPD, which confirms the theory that oxidative stress plays a role in BPD occurrence. Hence, valproate and lithium, which are the most commonly used mood stabilizers in the treatment of BPD, were shown to have neuroprotective effects when oxidative stress was induced in rat brains (Cui et al., 2007; Shao et al., 2008). It has been reported that chronic treatment with those drugs results in an increased expression of cellular glutathione S-transferase (Wang et al., 2004). Additionally, treatment with N-acetylcysteine, a precursor of antioxidant glutathione, led to a significant improvement in the course of BPD treatment (Berk et al., 2008).

## Concluding remarks

Psychiatric disorders are highly prevalent worldwide and can have an early onset. This allows for substantial impairment for patients in both productive and social aspects of life, resulting in low level of education, work absenteeism, unemployment, social isolation, marital disruption, and the need for caregiving in many cases (Kessler et al., 1997, 1998; Wu et al., 2005; Hyman, 2008). One of the main underpins in psychiatry is the diagnosis, which relies entirely on a clinical evaluation when symptoms become evident. Although, when the disorder reaches this stage, it is usually already fully established, which indicates a higher severity in combination with less effective treatments (Saia-Cereda et al., 2017). The underlying pathophysiology of these disorders remains undetermined and studies aiming to help in early detection and early intervention could yield substantial improvements to the outcome of the disorders (Insel, 2010). For that reason, the use of quantitative proteomics to investigate disease-specific protein and pathway signatures can improve the understanding of psychiatric disorders (Filiou et al., 2011). The presence of metabolic alterations related to energy pathways have been recurrently implied as one of the physiological features of SCZ, BPD, and MDD (Shao et al., 2008). By collecting information acquired from patient postmortem brain proteomic research, with a focus on energy metabolism, we could establish molecular similarities among the disorders, in addition to highlighting which pathways were most affected in each one. This study highlights the importance of the connection between psychiatrists and researchers to facilitate access to patient samples and stimulate a more comprehensible knowledge base acquired in this field. Consequently, the constant update and increase of data deposited in postmortem brain banks will contribute to a better comprehension of the pathophysiological mechanisms of psychiatric disorders, which can in turn improve the diagnosis, treatment, and potential to overcome these conditions, resulting in improvements in quality of life for the patients.

## Author contributions

GSZ acquired data from the literature and interpreted them, wrote the manuscript, and produced the figures. VMSC collected the data, assisted in their interpretation, and revised the manuscript. JMN assisted in interpreting the data, in elaborating the figures, writing the manuscript and revising the text. DMS contributed to the design of the work, assisted in interpreting the data, writing the manuscript, and revising the text. All four authors revised and approved the final version to be submitted.

### Conflict of interest statement

The authors declare that the research was conducted in the absence of any commercial or financial relationships that could be construed as a potential conflict of interest.

## Supplementary material

Supplementary Table 1 List of proteins related to energy metabolism found altered in SCZ, BPD and MDD.

Supplementary Table 2 Compilation of demographic and clinical data for the samples used in the different studies.
